# Do business strategies and environmental, social, and governance (ESG) performance mitigate the likelihood of financial distress? A multiple mediation model

**DOI:** 10.1016/j.heliyon.2023.e17847

**Published:** 2023-07-07

**Authors:** Ahmed Mohamed Habib

**Affiliations:** Accounting and Finance, Independent Research, Zagazig, Egypt

**Keywords:** Business strategy, Cost leadership, ESG performance, Environment, Governance, Social responsibility, Financial distress, Mediation analysis, Multivariate analysis, United States

## Abstract

This study explores the connection between business strategies, ESG performance, and the probability of bankruptcy. Using a sample comprising 1970 U.S. firm-year observations from 2016 to 2020, this study adopts several techniques to achieve its goals, including the partial least squares structural equation modeling (PLS-SEM) algorithm and additional analyses. The results demonstrate that a firm with a better cost leadership strategy has higher ESG performance. A sound cost leadership strategy and ESG performance negatively influence a firm’s likelihood of financial distress. Using a mediating analysis model, we also find that financial and ESG performance mediate and mitigate the probability of experiencing financial distress through a cost leadership strategy, indicating that these are essential factors that cannot be ignored when mitigating bankruptcy probability. Financial performance also mediates and mitigates the probability of experiencing financial distress through the ESG path. This study adds to the existing body of knowledge by revealing the role of sound business strategies and ESG performance in mitigating the likelihood of financial distress, an under-explored topic. It also analyzes the mediation roles of financial and ESG performance to provide significant insights to companies' decision-makers in order to support them in their endeavors toward performance improvement and achieving best practices.

## 1 Introduction

Business strategy is a necessary attribute that contributes to a firm’s success. A manager unaware of the essence and importance of a business strategy cannot guarantee the long-term sustainability of a firm [[1], [2], [3]]. Generally, a firm that desires excellence and sustainability needs a business strategy that is developed and applied to all its activities [4,5]. A business strategy is defined as a collection of decisions and guiding principles that help managers achieve a firm's goals [5] and is also described as a tool for realizing a firm's sustainability and vision for the future [[6], [7], [8], [9]]. In addition, business strategy is defined as an integrated and coordinated collection of commitments and actions to capitalize on core skills to achieve competitive advantages. In this sense, business strategy is deliberated upon to enhance the execution of guided activities [10].

A business-level strategy uses core competencies in specialized, individual product markets to enhance a firm's performance and deliver customer value [11]. Thus, firm-level strategy represents the perception of where and how a firm has an advantage over its competitors. Considering this philosophy, business strategy is aligned with the theory of the firm, as the firm is seen as a cluster of complementary attributes, including the concentration of decision rights over productive assets, regulation of managerial hierarchies, employment contracts, and the use of incentives and implicit agreements to regulate transactions [12]. From this perspective, a firm can efficiently allocate resources to enhance its performance and achieve competitive advantages [[13], [14], [15]]. In addition, there are numerous other conceptualizations of the nature of the firm, some based on notions of firms as specialized collections of heterogeneous resources [[16], [17], [18]], some based on behavioral theory [19,20], while others emphasize the nature of firms as knowledge-based and learning entities [[21], [22], [23]]. Thus, a better business strategy supports the firm's theory. Hence, a meaningful question to investigate is whether business strategy impacts performance, especially sustainability performance, and the possibility of mitigating potential risks, particularly, financial distress.

Refs. [24,25] identify three generic strategies for attaining competitive advantage: differentiation, cost leadership, and focus. According to Refs. [24,25], cost leadership entails firms providing products at lower costs and prices than their competitors, thereby gaining a competitive advantage. This strategy involves acquiring market share by appealing to cost-conscious and price-sensitive customers. This is accomplished by setting the lowest price or the price-to-value ratio in the desired market segment. Porter's model posits that a firm's success reflects its competitiveness. Therefore, firms with better business strategies may have a competitive advantage over their industry rivals [5]. Recent research contends that a firm can establish a competitive cost leadership strategy and generate superior performance using Porter's methodology [26,27]. In addition, experts emphasized the possibility of the plan's alignment with the continual improvement process during its development. Continuous improvement supports the successful execution of the chosen method [[28], [29], [30]]. When efficiently implemented and linked with other operational areas for constant improvement, firm-level business strategy methodologies exhibit substantial performance and cultural enhancement [31]. Consequently, several authors have advocated connecting continuous improvement strategies to competitive tactics [[32], [33], [34], [35], [36]].

Business strategies share some similarities with firms' ESG practices in that the purpose of adopting them is to achieve continuous improvement in the firm's business, whether in an internal or external context, to enhance performance, attract investors, and gain a competitive advantage. ESG is a business strategy that enhances environmental, social, and governance practices [37,38]. Firms that integrate sustainable practices into their operations experience better performance and market value [[37], [38], [39], [40], [41], [42], [43]]. Ref. [44] confirm that sustainability performance practices positively affect firm efficiency. Refs. [37,45,46] confirm that better ESG practices are required to reduce risk. Several theories have been proposed to explain this issue, including stakeholder, institutional legitimacy, signaling, and agency theories [39,[47], [48], [49], [50], [51]].

Moreover, accounting and business research concentrates on detecting potential indicators or red flags instead of determining direct causes or antecedents [52]. Business strategy and ESG practices, as essential factors affecting firm performance [37,53,54] have received little attention regarding their impact on firms' probability of experiencing financial distress. Our study explores whether business strategies and ESG practices are essential to mitigate firms' likelihood of experiencing financial distress. Our study provides evidence that increases our understanding of the underlying determinants of the probability of experiencing financial distress, thus helping decision-makers enhance their strategies, improve ESG performance, and help investors allocate their funds to less risky businesses.

This study explores the relationship between firms' business strategies, ESG performance, and the probability of financial distress. In this study, we use the cost leadership strategy axis proposed by Refs. [24,25] to examine whether firms that follow this strategy exhibit higher ESG performance and lower probability of bankruptcy. Additionally, we examine whether firms with better ESG performance have lower bankruptcy potential. Furthermore, we investigate the interactions of ESG and financial performance with a cost leadership strategy to ascertain whether these factors mediate the relationship between a cost leadership strategy and the probability of financial distress.

In addition, we use U.S. companies from 2016 to 2020 to analyze the connections between business strategies, ESG performance, and the probability of bankruptcy. The current study has distinctive attributes and motives in the context of U.S. companies. First, ESG practices are essential for improving the performance of U.S. firms [37,38]. Second, a McKinsey report indicated that ESG investments increased as inflow investments into firms' sustainable funds rose from $5 billion to $50 billion, especially from 2018 to 2020 [55]. Such an increase burdens firms' financial resources, requiring a return assessment of these investments and adopting appropriate measures to reveal deficiencies or deviations in ESG practices. Third, firms listed on U.S. stock markets face more pressure to meet financial and market targets, although the U.S. economy faces inflationary pressure. Because stagflation hazards increase amid an intense downshift in growth, worldwide growth is predicted to decrease from 5.7% in 2021 to 2.9% in 2022 [56]. The U.S. economy is suffering from a stagflation crisis, and U.S. businesses must prepare for this reality by developing business cost leadership strategies to reduce spending and enhance overall performance. Finally, given the shocks provoked by the coronavirus crisis and Russia's attack on Ukraine, crises could worsen for firms and economies in the production process and provide the necessary resources, that is, supply chain security. Therefore, U.S. firms need to improve their business strategy to make the best possible use of their resources and improve their performance to reduce the likelihood of financial failure.

Our results support our forecasts and demonstrate an important connection between business strategies, ESG performance, and likelihood of experiencing financial distress. The results demonstrate that firms' business strategies influence their ESG performance. Specifically, we find that firms that follow cost leadership business strategies have better ESG performance and are slightly more likely to experience financial distress. Using the mediating analysis model, we also find that financial and ESG performance mediate and mitigate the probability of experiencing financial distress through firms' cost leadership strategies, thereby strengthening the adverse connection between firms' cost leadership strategies and the probability of bankruptcy. Financial performance also mediates and mitigates the probability of experiencing financial distress through the ESG path, indicating that this is an essential factor that cannot be ignored when mitigating the bankruptcy probability.

The remainder of this paper is organized as follows: Section 2 reviews the relevant literature. Section 3 describes the data and the methodology used in this study. Section 4 presents the empirical results. Section 5 discusses the findings, and Section 6 concludes the study.

## 2 Theoretical background

Contingency theory highlights the significance of a manager's personality and the conditions in which he operates, as effective leadership relies on the type of leadership and control over the situation. There is a need for sound leader–member links, tasks with transparent plans and policies, and the capacity of the manager to ration punishments and rewards [[57], [58], [59]]. Ref. [60] argued that top leaders' knowledge is significant in leading a firm's strategy and affecting strategic outcomes. If a business strategy is developed, its consequences will be integral and evident. Refs. [58,59,61] argued that optimal business strategy is critical for directing a firm's activities and improving overall productivity and performance. This study argues within the identical context that business strategies, such as those connected to cost leadership, are strategic decisions that managers make and would probably influence ESG performance and mitigate potential risks. The evolutionary theory emphasizes the importance of a firm evolving into an ecosystem of continual creativity [62]. This evolutionary approach involves building a substantial variation from which reasonable practices can be selected and retained to achieve competitive advantage [[62], [63], [64]]. In the same context, this study argues that a firm's evolution cannot occur without sound business strategies or efficient ESG practices. As one of the most noteworthy theories in business, the resource-based theory was conceived to clarify the sources of sustainable competitive advantage in firms [16,38,65,66]. According to the resource-based view, firms' internal capabilities and resources are the most suitable sources for enhancing performance and achieving competitive advantage [67]. Similarly, this study argues that firm resources cannot be well managed without sound business strategies.

In addition, several theories have been proposed to explain this issue, including stakeholder, institutional legitimacy, signaling, and agency theories. The stakeholder theory assumes that satisfying stakeholders' needs are crucial challenge concerning information asymmetries and conflicts of interest between stakeholders [38,51]. It states that a firm's sustainability practices can help enhance its connections with stakeholders and its ability to attract investors [49,50,68,69]. Ref. [51] confirms that institutions and society are intertwined according to institutional legitimacy theory because there is congruence between the institution's acts and the shared and supposed views of the relevant social groupings. Thus, the intertwined relationship between society and the institution implies that the institution provides valuable and desirable products or services to the community and distributes products or services to customers fairly. In addition, Ref. [48] suggest that, based on institutional legitimacy theory, investing in sustainability performance, regardless of size, improves financial performance. As a result, good ESG practices would enhance the relationship and better meet these demands [39]. Moreover, signaling theory helps describe the behavior of firms in society. Sustainability practices refer to how firms convey information regarding their abilities. Refs. [47,70] indicate that, according to signaling and agency theories, managers of profitable companies are more likely to voluntarily deliver influential information in their annual financial reports to signal their companies' profitability and boost investor confidence and compensation. Refs. [71,72] confirm that ESG disclosures can enhance a firm's reputation and mitigate potential risk.

Therefore, this study explores the role of business strategy in enhancing ESG performance and mitigating potential financial risks in light of theories of contingency, evolution, resources, stakeholder engagement, institutional legitimacy, signaling, and agency.

## 3 Literature review and hypotheses development

### 3.1 Business strategies and ESG performance

In the managerial literature, business strategy is a significant attribute that supports a firm's success and long-term sustainability [[1], [2], [3],^7^,^9^]. It focuses on how to compete in a business environment and is a source of intra-industry variation [5,[73], [74], [75]]. Business strategy reflects the actions and choices taken by a firm to enhance its performance and deliver value to its customers [11].

The literature contains several business strategy taxonomies describing how companies compete in specific industries. In this study, we use the business strategy axis proposed by Refs. [24,25] as a cost leadership strategy to examine whether firms that follow this strategy exhibit higher ESG performance and lower probability of experiencing bankruptcy. In the context of this axis, a cost leadership strategy entails firms producing products at a lower cost and price than their competitors, thereby gaining competitive advantage. This strategy involves gaining market share by appealing to cost-conscious and price-sensitive customers. Generally, firms with better business strategies have a competitive advantage over their industry rivals [5]. Ref. [76] examined the connection between firms' business strategies and sustainability performance from a corporate social responsibility (CSR) perspective from 2004 to 2012, and the results suggest that firms' business strategies significantly determine their sustainability performance. Ref. [77] investigated the linkage between business strategies and sustainability performance in CSR initiatives by utilizing a set of Chinese manufacturing firms, and the results indicate that business strategies can improve the performance of sustainability practices. Recent research contends that a firm can establish a competitive cost leadership strategy and generate superior performance using Porter's methodology [26,27].

Business strategies delineate firms' plans, activities, and decisions to accomplish their objectives and goals. These strategies reveal how firms plan to place themselves in the market. In the context of contingency theory, Ref. [60] argued that managers' experience is essential for directing a firm's strategy, which affects strategic outcomes. Refs. [58,59,61] argued that business strategies are essential in directing a firm's strategy, affecting overall productivity, and leading to performance improvement. This study argues within the identical context that business strategies such as those connected to cost leadership are managers' strategic decisions and may influence ESG performance. Additionally, from the perspective of evolutionary theory, a firm's ecosystem development is an important issue [62]. In the same context, this study argues that a firm's evolution cannot occur without sound business strategies and efficient ESG practices. From a resource-based perspective, firms' internal capabilities and resources are the most suitable sources for enhancing performance and achieving competitive advantage [67]. Resource-based theory states that a firm competes with others based on its capabilities and resources. If a firm employs its capabilities and resources well, it can achieve superior performance and enjoy sustainable competitive advantages [16,28,29,78]. In the same context, this study argues that firm resources cannot be managed well without sound business strategies and efficient ESG practices. From a broad perspective, business strategies indicate how a firm makes value for all its shareholders, legitimizes and signals its presence, reduces agency conflicts, enhances its value, and secures its sustainability. Sustainability performance has long been considered a practical path to supporting firms in developing capabilities and resources that guide competitive advantage [[48], [49], [50],^68^,^69^], ESG practices can improve firms' fostering of customer trust and reputation, thereby contributing to resource development [47,71,72]. Based on prior literature, theories, and theoretical business strategy frameworks [24,25], this study argues that firms' business strategies are linked to their ESG performance. Therefore, the following hypothesis is formulated:

H1 Firms' business strategies positively affect ESG performance.

### 3.2 Business strategies and the likelihood of financial distress

Refs. [24,25] indicated that a firm's success depends on its competitiveness. Therefore, firms with better business strategies may have a competitive advantage over their industry rivals [5]. Recent research contends that a firm can establish a competitive cost leadership strategy and generate superior performance using Porter's methodology [26,27]. According to Refs. [24,25], a cost leadership strategy entails firms producing products at a lower cost and delivering them at a lower price than their competitors, thereby gaining competitive advantage. This strategy involves gaining market share by appealing to cost-conscious and price-sensitive customers. This is accomplished by setting the lowest price or the price-to-value ratio in the desired market segment. The cost leadership strategy is mainly oriented toward productivity optimization by adopting efficient initiatives for managing costs without prejudice to the product's quality and optimizing the usage of firm assets in the production process [79]. Hence, a cost leadership approach is intrinsically tied to productivity enhancements [79] through the efficient integration of various inputs to maximize production outputs. Ref. [80] emphasized that firms innovate and strategically employ newer technologies to achieve competitive and other goals. Ref. [81] confirmed that optimal business strategy is the primary source for boosting firm performance. Firms can employ various procedures to accomplish cost leadership initiatives, such as target costing, large-scale production to fulfill thrifts of scale, benchmarking, total quality management, just-in-time processes, and statistical process control [82]. Ref. [79] confirmed that firms with better business strategies are more robust when facing bankruptcy risk. Based on prior literature ensuring that sound performance can mitigate insolvency hazards [29,83], theories, and theoretical business strategy frameworks [24,25], this study argues that a firm's business strategies may play an influential role in mitigating the likelihood of financial distress. Therefore, the following hypothesis is formulated:

H2 Firms' business strategies negatively affect the likelihood of experiencing financial distress.

### 3.3 ESG performance and the likelihood of financial distress

In the context of sustainability, experts emphasized the possibility of the plan's alignment with the continual improvement process during business strategy development. Continuous improvements support the successful execution of the selected method [28,29]. When efficiently implemented and linked with other operational areas for constant improvement, firm-level business strategy methodologies have shown substantial performance and cultural enhancements [31]. Consequently, several authors have advocated connecting continuous improvement strategies to competitive tactics [[32], [33], [34], [35], [36]]. Business strategies share some similarities with firms' ESG practices in that the purpose of adopting them is to achieve continuous improvement in the firm's business, whether in the internal or external context, to enhance performance, attract investors, and gain a competitive advantage. ESG is a business strategy that enhances sustainability practices [37]. Firms that integrate sustainable practices into their operations experience better performance and value [37,[39], [40], [41], [42], [43]]. Ref. [84] indicated that sustainability practices increase client loyalty and provide firms with competitive advantages. Based on the literature confirming that sustainability performance effectively supports firms in developing capabilities and resources that guide competitive advantage [[48], [49], [50],^68^,^69^] and the literature demonstrating that firms' ESG practices can reduce risks [37,45,46], the following hypothesis is formulated:

H3 Firms' ESG performance negatively affects the likelihood of experiencing financial distress.

### 3.4 Mediation perspective

Business strategies share some similarities with firms' ESG practices in that the purpose of adopting them is to achieve continuous improvement in the firm's business. In this regard, [85] examined the relationship between ESG and financial distress using a sample of 362 U.S. commercial banks from 2012 to 2019. They found that ESG strongly reduces the likelihood of financial distress. Ref. [86] investigated the influence of ESG parameters on companies' financial stability using a sample of 691 firms in North American countries from 2011 to 2020. They conclude that ESG factors positively impact firms' financial success. Ref. [87] investigated the relationship between CSR and the probability of bankruptcy using a sample of Tehran firms from 2009 to 2016. They find that CSR is important in reducing the probability of firm bankruptcy. Ref. [88] investigated the relationship between CSR disclosure levels and bankruptcy risk using a sample of 63 Vietnamese firms from 2014 to 2018. They find that firms with higher levels of CSR disclosure can rapidly reduce their bankruptcy risk. In addition, the results also indicate a difference in bankruptcy risk between firms that disclose CSR and those that do not disclose CSR in their annual reports. Ref. [89] examined the relationship between CSR and corporate bankruptcy using a sample of 78 publicly traded U.S. firms from 2007 to 2014. They indicate that firms with more robust CSR are less likely to become bankrupt than those with weaker CSR. Ref. [90] investigated whether CSR engagement could be a risk mitigation instrument. The study reveals that the initiatives of CSR aid in expediting a company's recovery from insolvency and enhances the probability of a financially troubled firm negotiating with financial financiers effectively. Hence, to further investigate the link between business strategies and the probability of bankruptcy and after considering the findings that verify that better performance and strategies lead to lower bankruptcy risk [29,79,83], this study argues that a firm's ESG and financial performance are significant in the connection between firms' business strategies and the probability of bankruptcy. Therefore, the following hypotheses are formulated:

H4 ESG performance mediates the relationship between firms' business strategies and likelihood of financial distress.

H5 Financial performance mediates the relationship between firms' business strategies and likelihood of financial distress.

For more investigation of the link between ESG performance and the probability of bankruptcy, and after considering the findings that verify that better performance leads to lower risk [29,83], this research argues that a company's financial performance is significant in the connection between firms' ESG performance and the probability of bankruptcy. Therefore, the following hypothesis is formulated:

H6 Financial performance mediates the relationship between ESG performance and likelihood of financial distress.

## 4 Methodology

### 4.1 Data description

A significant part of ESG investment growth is driven by responses to climate change and the desire to achieve comprehensive, outstanding performance in the environmental, social, and economic fields, and to enhance governance performance, thereby helping firms mitigate the probability of experiencing bankruptcy. The association of business strategy and ESG performance with the likelihood of default is clarified through data gathering for U.S. firms recorded on the NASDAQ-CM and NYSE. Financial data were collected from Standard & Poor's platform from 2016 to 2020. The initial sample includes 2090 firm-year observations from 418 U.S. firms. As a result of the shortage of data for some firms, our final selection comprises 1970 firm-year observations in longitudinal panel data from 394 U.S. firms.

### 4.2 Research design

**Fig. 1:**
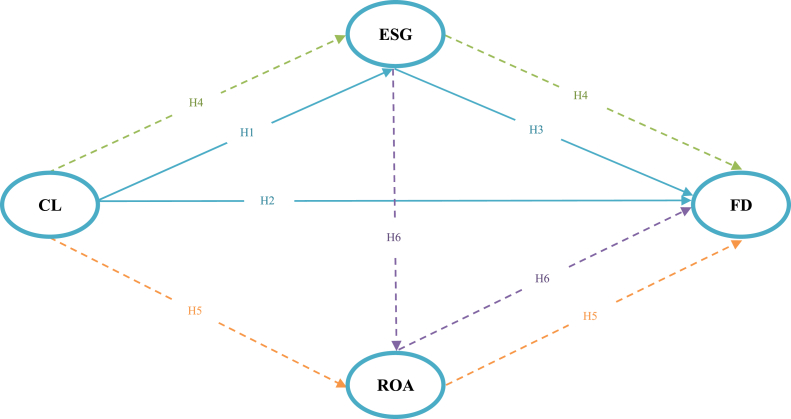
Conceptual Model. Note: The variables include cost leadership (CL), environmental, social, and governance (ESG), return on assets (ROA), and financial distress (FD).  represents the direct impact;  represents the indirect impact.

This study explores the impact of business strategy and ESG performance on the probability of bankruptcy. The mediating effects were also considered. We employed the PLS-SEM algorithm to accomplish our study goals. The conceptual model of the framework used in this study is shown in Fig. 1.

In the context of the conceptual model of the framework and to verify the connection between business strategy and ESG performance, we constructed the following econometric model:

(1) ESG_i,t_ = β_0_ + β_1_CL_i.t_ + β_n_∑CON_i,t_+ ℇ_i,t_

where *i* represents firms, *t* represents years, and the dependent variable *(ESG*_*i.t*_*)* indicates a firm's environmental, social, and governance performance. *CL*_*i.t*_ indicates a firm's cost leadership level. *CON*_*i,t*_ contains a series of firm-level control variables such as firm size *(SIZE),* leverage *(LEV),* firm age *(AGE)*, the coronavirus crisis *(COV),* and sector classification *(SEC)*. *ℇ*_*i,t*_ is the random error term. If the estimation coefficient *β*_*1*_ of *CL* is significantly positive, thus indicates that the cost leadership strategy enhances the ESG performance. See Table 1 for specific definitions of each variable.

**Table 1:** Variables definition.

Variable	Definition
Financial distress (FD)	*FD* is the likelihood of a firm’s financial distress, calculated by the Altman Z-score bankruptcy likelihood model and expressed by a dummy variable taking *zero* for the safe zone and *one* otherwise.
Cost leadership (CL)	*CL* is the natural log of a firm’s operating sales to its operating assets at the end of year *t*.
Environmental, social, and governance (ESG)	*ESG* is the natural log of a firm’s environmental, social, and governance performance at the end of year *t*, collected from the Standard & Poor’s platform.
Firm size (SIZE)	*SIZE* is the natural logarithm of a firm’s total assets at the end of year *t*.
Leverage (LEV)	*LEV* is the natural logarithm of a firm’s debt to total assets at the end of year *t*.
Firm age (AGE)	*AGE* is the natural logarithm of the years from establishment to the end of year *t*.
Return on assets (ROA)	*ROA* is the natural log of a firm’s net income to its total assets at the end of year *t*.
Coronavirus crisis (COV)	*COV* is the coronavirus crisis, calculated by a dummy variable taking *one* for the time of the crisis and *zero* otherwise.
Sector classification (SEC)	*SEC* is the Sector classification, calculated by a dummy variable taking *one* for manufacturing firms and *zero* for non-manufacturing and service firms.

In the context of the conceptual model of the framework used and to verify the role of mediation variables, we constructed the following econometric model:

(2) FD_i,t_ = β_0_ + β_1_CL_i.t_ + β_2_ESG_i,t_ + β_3_ROA_i,t_ + β_4_(ESG_i,t_*CL_i,t_) + β_5_(ROA_i,t_*CL_i,t_) + β_6_(ROA_i,t_*ESG_i,t_) + β_n_∑CON_i,t_ + ℇ_i,t_

where *i* represents firms, *t* represents years, and the dependent variable (*FD*_*i,t*_) denotes the probability of bankruptcy calculated by the Altman *Z-score* bankruptcy likelihood model. As introduced in previous studies, the *Z-score* can be expressed as a linear discriminant function of the five financial ratios in a multivariate context, defined as follows:

(3) Z = 1.2X_1_ + 1.4X_2_ + 3.3 X_3_ + 0.6 X_4_ + 0.99 X_5_

where *X*_*1*_ is the percentage of working capital to total assets, *X*_*2*_ is the percentage of retained earnings to total assets, *X*_*3*_ is the percentage of earnings before interest and taxes to total assets, *X*_*4*_ is the percentage of the market value of equity to total liabilities, and *X*_*5*_ is the percentage of sales to total assets. According to the *Z-score*, a firm is in the safe zone for values greater than 2.99 and in the distress zone for values lower than 1.81. The intermediate values between these two extremes represent the so-called “gray area”, which signals uncertainty regarding a firm's viability.

The Altman *Z-score* bankruptcy likelihood model is one of the best-known distress prediction models [29,79,83,89,[91], [92], [93]]. In addition, Ref. [94] evaluated the validity of the Altman model's predictive power over the past 50 years since it was first formulated in 1968. The results showed remarkable flexibility and high predictive ability for bankruptcy over the years, despite the tremendous increase in the size and complexity of worldwide debt markets and firms' balance sheets.

The explanatory variable (*CL*_*i.t*_*)* indicates a firm's cost leadership strategy level. *ESG*_*i.t*_ indicates a firm's ESG performance. *ROA*_*i.t*_ indicates a firm's return on assets. The mediating explanatory variable *(ESG*_i,t_*CL_*i,t*_*)* reflects the mediating role of ESG performance in the connection between a firm's cost leadership strategy and the likelihood of financial distress; *(ROA*_i,t_*CL_*i,t*_*)* reflects the mediating role of return on assets in the connection between a firm's cost leadership strategy and the likelihood of financial distress; and *(ROA*_i,t_*ESG_*i,t*_*)* reflects the mediating role of return on assets in the connection between a firm's ESG performance and the likelihood of financial distress. *CON*_*i,t*_ contains a series of firm-level control variables such as firm size *(SIZE),* leverage *(LEV),* firm age *(AGE)*, coronavirus crisis *(COV),* and sector classification *(SEC). ℇ*_*i,t*_ is the random error term. If the estimation coefficients *β*_*1*_ and *β*_*2*_ of *CL* and *ESG* are significantly negative, then the cost leadership strategy and ESG performance mitigate the likelihood of bankruptcy. See Table 1 for specific definitions of each variable.

For further analysis, robustness tests were performed to ascertain the robustness of the findings using alternative estimations. We used different strategies to verify whether they resulted in substantial differences in the results, such as the logistic regression approach, logistic regression estimations with robust standard errors, and logistic regression estimations using a bootstrapping technique with 5000 replications. Accordingly, we construct the following econometric model:

(4) FD_i,t_ = β_0_ + β_1_CL_i.t_ + β_2_ESG_i,t_ + β_3_∑α_i_ + β_4_∑γ_t_ + β_n_∑CON_i,t_ + ℇ_i,t_

In Model (4), *i* represents firms, *t* represents years, and the dependent variable (*FD*_*i,t*_) denotes the probability of bankruptcy. It is expressed by a dummy variable that takes the value of *zero* for the safe zone and *one* otherwise. *CL*_*i.t*_ indicates a firm's cost leadership level. *ESG*_*i.t*_ indicates a firm's ESG performance. *α*_*i*_ represents industry-fixed effects, and *γ*_*t*_ represents year-fixed effects. *CON*_*i,t*_ contains a series of firm-level control variables such as firm size *(SIZE),* leverage *(LEV),* firm age *(AGE)*, firm return on assets *(ROA),* coronavirus crisis *(COV),* and sector classification *(SEC). ℇ*_*i,t*_ is the random error term. See Table 1 for specific definitions of each variable.

## 5 Empirical results

### 5.1 Descriptive statistics

Table 2 presents the descriptive statistics for the key variables. The FD of firms ranges between 0 and 1, with a mean of 0.502, suggesting that approximately 49.8% of firm-year observations are in the safe zone of the probability of distress, and approximately 50.2% of firm-year observations are in the gray and distress zones. The firms' ESG and CL averages are 3.305 and 3.568, respectively, with minimum and maximum values of 1.386–4.466 and −2.210–5.393, respectively. This reflects the relatively high level of ESG performance and cost leadership strategy practices in U.S. firms. The means of SIZE, LEV, and AGE are approximately 9.839, 0.716, and 4.098, respectively, with a minimum of 5.280, −2.618, and 0 and a maximum of 15.04, 4.534, and 5.464, respectively, indicating a relative discrepancy in size, leverage, and age between firms. The mean ROA is around −3.155, with a range between a minimum of −7.706 and a maximum of −1. The COV and SEC averages are 0.2 and 0.152, respectively, with a minimum and maximum of 0–1. This study conducted a variance inflation factor test to check for multicollinearity among variables. The test results reveal that the VIF values of all variables were significantly lower than 5, indicating the absence of collinearity among the variables selected in this study.

**Table 2:** Descriptive statistics summary.

Variables	Obs	Mean	Std. dev.	Min	Max
FD	1970	0.502	0.500	0.000	1.000
CL	1970	3.568	1.255	−2.210	5.933
ESG	1970	3.305	0.584	1.386	4.466
SIZE	1970	9.839	1.420	5.280	15.04
LEV	1970	0.716	1.034	−2.618	4.534
AGE	1970	4.098	0.750	0.000	5.464
ROA	1970	−3.155	1.004	−7.706	−1.000
COV	1970	0.200	0.400	0.000	1.000
SEC	1970	0.152	0.359	0.000	1.000

Note: The variables include financial distress *(FD),* environmental, social, and governance *(ESG),* cost leadership *(CL),* firm *size (SIZE),* firm leverage *(LEV),* firm age *(AGE),* return on assets *(ROA),* coronavirus crisis *(COV),* and sector classification *(SEC).*

### 5.2 Analysis of discriminant validity

Table 3 presents the discriminant validity of the variables used in this study using the Fornell-Larcker criterion. Panel A shows that CL and ROA correlate significantly and negatively with FD. This result signifies that a higher-cost leadership strategy and better financial performance mitigate the probability of bankruptcy. SEC was also significantly and negatively correlated with FD. This result signifies that manufacturing companies are unlikely to experience bankruptcy. In contrast, SIZE and LEV were significantly positively correlated with FD. This finding indicates that larger companies and those with higher financial leverage are more likely to experience bankruptcy. COV was also significantly and positively associated with FD. This result signifies that the probability of default was higher during the coronavirus crisis. CL correlates significantly and positively with ROA and SEC. This indicates that companies with a higher-cost leadership strategy have better financial performance and are characterized as manufacturing companies. Conversely, CL significantly negatively correlated with SIZE, LEV, and AGE. This result signifies that companies with a higher-cost leadership strategy are distinguished as being smaller in size, leverage, and age. In addition, the results demonstrated that CL was significantly and negatively correlated with COV. This result signifies that companies' cost leadership strategies were better before the coronavirus crisis than during the crisis period. ESG was significantly positively correlated with SIZE and LEV. This result signifies that companies with higher ESG performance are larger and more leveraged. These results highlight the need to balance companies' resources and working capital to achieve optimal levels of financial leverage, which ultimately contributes to achieving desired goals and is inherently reflected in performance measures. The discriminant validity ratios are below the threshold of 0.85. Therefore, we can conclude that the measurement model is satisfactory. Furthermore, the results in Panel B show the absence of any issues pertaining to multicollinearity during the computation of the VIF, as the maximum value was 2.27, and the variables' tolerance values also exhibited a range of 0.440–0.990. Thus, no multicollinearity concerns exist among the latent variables [95,96].

**Table 3:** Analysis of discriminant validity.

Panel A: Discriminant validity through Fornell-Larcker criterion									
Variables	FD	CL	ESG	SIZE	LEV	AGE	ROA	COV	SEC
FD	1.000								
CL	−0.488***	1.000							
ESG	−0.034	0.019	1.000						
SIZE	0.140***	−0.128***	0.101***	1.000					
LEV	0.480***	−0.326***	0.083***	0.176	1.000				
AGE	0.032	−0.038*	0.013	0.019	0.112***	1.000			
ROA	−0.462***	0.351***	0.013	−0.106	−0.373***	−0.029	1.000		
COV	0.070***	−0.048**	0.000	0.034	0.031	0.000	−0.047**	1.000	
SEC	−0.222***	0.201***	−0.001	−0.074**	−0.092***	0.023	0.120***	0.000	1.000
Panel B: Multicollinearity test									
Criterion		ESG	CL	SIZE	LEV	AGE	ROA	COV	SEC
VIF		1.22	2.19	1.74	1.76	1.12	2.27	1.01	1.11
Tolerance		0.818	0.456	0.576	0.569	0.889	0.440	0.990	0.904

Note: *, **, and *** denote significance at the *10%, 5%,* and *1%* levels, respectively. This table reports the discriminant validity of the variables used in this study through the Fornell-Larcker criterion. The variables include financial distress *(FD),* environmental, social, and governance *(ESG),* cost leadership *(CL),* firm *size (SIZE),* firm leverage *(LEV),* firm age *(AGE),* return on assets *(ROA),* coronavirus crisis *(COV),* and sector classification *(SEC).* The discriminant validity ratios are below the threshold of *0.85*. Therefore, we can conclude that the measurement model is satisfactory. Furthermore, the results showed no multicollinearity problems when calculating the variance inflation factor *(VIF),* as the highest value was *2.27*. Similarly, the tolerance values of the variables ranged from *0.440* to *0.990*. Thus, there was no multicollinearity concern among the latent variables.

### 5.3 Multivariate analyses

**Fig. 2:**
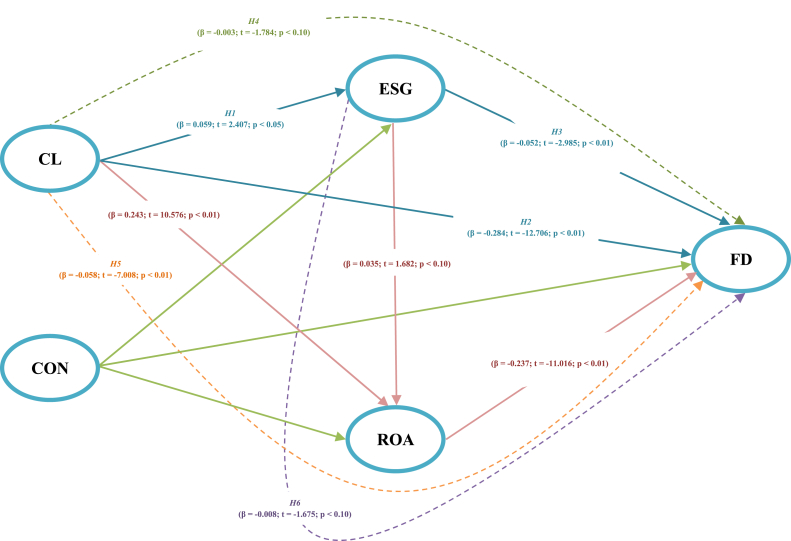
Bootstrapping of the structural model. Note: The variables include financial distress (FD), environmental, social, and governance (ESG), return on assets (ROA), cost leadership (CL), and control variables (CON) which include firm size (SIZE), firm leverage (LEV), firm age (AGE), coronavirus crisis (COV), and sector classification (SEC). H4 represents the “CL -> ESG -> FD” path; H5 represents the “CL -> ROA -> FD” path; H6 represents the “ESG -> ROA -> FD” path.

Using the PLS-SEM algorithm for multivariate analyses, this study is conducted using the PLS algorithm and the Smart PLS 3 software. Fig. 2 shows bootstrapping of the structural model.

Bootstrapping of the structural model was conducted employing 5000 bootstrap replications and a 95% confidence range. As shown in Fig. 2, CL significantly and positively influences ESG and financial performance. In addition, CL and ESG performance significantly and negatively affected the FD. From the perspective of evolutionary theory, this study argues that a firm’s evolution cannot occur without sound business strategies and efficient ESG practices. Table 4 shows the results of the path coefficients, while Table 5 summarizes the paths and mediation effects of the research hypotheses.

**Table 4:** Paths coefficients results.

Relationship	β	Std. err.	T-value	P-value	Confidence Intervals	
					Lower	Upper
CL -> ESG	0.059	0.024	2.407**	0.016	0.013	0.109
CL -> FD	−0.284	0.022	−12.706***	0.000	−0.329	−0.240
CL -> ROA	0.243	0.023	10.576***	0.000	0.197	0.288
ESG -> FD	−0.052	0.017	−2.985***	0.003	−0.086	−0.018
ESG -> ROA	0.035	0.021	1.682*	0.093	−0.006	0.074
ROA -> FD	−0.237	0.021	−11.016***	0.000	−0.280	−0.195
SIZE -> ESG	0.093	0.024	3.901***	0.000	0.046	0.139
SIZE -> FD	0.024	0.017	1.439	0.150	−0.010	0.057
SIZE -> ROA	−0.024	0.022	−1.075	0.283	−0.068	0.019
LEV -> ESG	0.086	0.025	3.497***	0.000	0.036	0.133
LEV -> FD	0.290	0.019	15.014***	0.000	0.252	0.327
LEV -> ROA	−0.289	0.024	−12.093***	0.000	−0.335	−0.242
AGE -> ESG	0.003	0.021	0.160	0.873	−0.037	0.045
AGE -> FD	−0.016	0.017	−0.905	0.366	−0.049	0.018
AGE -> ROA	0.012	0.021	0.539	0.590	−0.033	0.052
COV -> ESG	−0.003	0.023	−0.115	0.908	−0.047	0.041
COV -> FD	0.035	0.018	1.935*	0.053	−0.001	0.070
COV -> ROA	−0.026	0.022	−1.193	0.233	−0.068	0.016
SEC -> ESG	0.002	0.023	0.066	0.947	−0.045	0.047
SEC -> FD	−0.108	0.020	−5.416***	0.000	−0.147	−0.070
SEC -> ROA	0.042	0.017	2.455**	0.014	0.008	0.077
CL -> ESG -> FD	−0.003	0.002	−1.784*	0.074	−0.007	−0.001
CL -> ROA -> FD	−0.058	0.008	−7.008***	0.000	−0.075	−0.043
ESG -> ROA -> FD	−0.008	0.005	−1.675*	0.094	−0.018	0.001

Note: *, **, and *** denote significance at the *10%, 5%,* and *1%* levels, respectively. This table summarizes the path coefficients using the Smart PLS 3 software. The variables include financial distress *(FD),* environmental, social, and governance *(ESG),* cost leadership *(CL),* firm *size (SIZE),* firm leverage *(LEV),* firm age *(AGE),* return on assets *(ROA),* coronavirus crisis *(COV),* and sector classification *(SEC).*

**Table 5:** Summary of paths coefficients, predictive accuracy, and validity measure.

Panel A: Paths coefficients of the research hypotheses						
Relationship	Hypo.	β	Std. err.	T-value	P-value	Decision
CL -> ESG	$H_{1}$	0.059	0.024	2.407	0.016	Supported**
CL -> FD	$H_{2}$	−0.284	0.022	−12.706	0.000	Supported***
ESG -> FD	$H_{3}$	−0.052	0.017	−2.985	0.003	Supported***
CL -> ESG -> FD	$H_{4}$	−0.003	0.002	−1.784	0.074	Supported*
CL -> ROA -> FD	$H_{5}$	−0.058	0.008	−7.008	0.000	Supported***
ESG -> ROA -> FD	$H_{6}$	−0.008	0.005	−1.675	0.094	Supported*

Panel B: Analyses of predictive accuracy and validity measures of the study model			
R-Square	R Square adjusted	Predictive accuracy (Geisser’s Q2 value)	Validity power (GoF value)
0.418	0.416	0.413	0.461

Note: *, **, and *** denote significance at the 10%, 5%, and 1% levels, respectively. This table summarizes the path coefficients, mediation effects, predictive accuracy, and validity measures of the research model using the Smart PLS 3 software. The variables include financial distress *(FD)*, environmental, social, and governance *(ESG)*, cost leadership *(CL)*, firm size *(SIZE)*, firm leverage *(LEV)*, firm age *(AGE)*, return on assets *(ROA)*, coronavirus crisis *(COV)*, and sector classification *(SEC)*. Coefficients of determination values denote moderate determination; blindfolding *(Geisser’s Q*^*2*^*)* values denote the predictive accuracy of all endogenous latent variables; the GoF value denotes the validity power *(high fitness)* of the study model as long as it is greater than *0.36*.

Table 5, Panel A, summarizes the path coefficients of the research hypotheses. As shown in Panel A, CL significantly and positively influences ESG performance (β = 0.059; t = 2.407; p < 0.05). Therefore, these results suggest that a firm with a better cost leadership strategy has better ESG performance, consistent with theoretical business strategy frameworks [24,25] and theories of contingency, evolutionary, resource-based, stakeholder, institutional legitimacy, signaling, and agency. Accordingly, these results support H1. These findings are consistent with those of previous studies [26,27,76,77]. CL significantly and negatively influenced FD (β = −0.284; t = −12.706; p < 0.01). These results indicate that a firm with a better cost leadership strategy is less likely to experience financial distress. Accordingly, these results support H2 predictions. These findings are compatible with prior theories, theoretical business strategy frameworks [24,25], and the literature, indicating that firms with better business strategies are more robust when facing bankruptcy risk [79]. ESG significantly and negatively influenced FD (β = −0.052; t = −2.985; p < 0.01). These results indicate that a firm with better ESG performance is less likely to experience bankruptcy. Accordingly, these results support H3. These findings are compatible with prior theories, theoretical business strategy frameworks [24,25], and the literature indicating that better ESG performance leads to a lower risk of insolvency [37,45,46]. ESG mediated the connection between CL and FD (β = −0.003, t = −1.784; p < 0.10). These results imply that better ESG performance strengthens the negative relationship between a firm’s cost leadership strategy and likelihood of experiencing financial distress. Accordingly, these results partially support H4. These findings are consistent with prior theories, theoretical business strategy frameworks [24,25], and the literature indicating that better ESG performance leads to a lower risk of insolvency [37,45,46]. Generally, firms that integrate sustainable practices into their operations experience better performance and value [37,[39], [40], [41], [42], [43]]. Ref. [84] suggested that sustainability practices increase client loyalty and provide firms with a competitive advantage. Similarly, ROA mediates the connection between CL and FD (β = −0.058, t = −7.008; p < 0.01). Therefore, these results imply that better financial performance strengthens the negative relationship between a firm’s cost leadership strategy and likelihood of experiencing financial distress. Accordingly, these results support H5. These findings are consistent with previous theories, theoretical business strategy frameworks [24,25], and the literature, which indicate that better performance leads to a lower risk of insolvency [29,37,45,46,83]. Furthermore, the outcomes reveal that ROA mediates the connection between ESG and FD (β = −0.008, t = −1.675; p < 0.10). These results imply that better financial performance strengthens the negative relationship between ESG performance and likelihood of experiencing financial distress. Accordingly, these results partially support H6. These findings are consistent with prior theories, theoretical business strategy frameworks [24,25], and the literature, which indicate that better performance leads to a lower risk of insolvency [29,37,45,46,83].

### 5.4 Predictive accuracy and validity measures

Table 5, Panel B, summarizes the analyses of the predictive accuracy and validity measures. Based on the instructions in Ref. [97], the results of the coefficient of determination and adjusted coefficient of determination for the study model indicated moderate variance. The predictive accuracy was estimated using blindfolding (Geisser’s Q^2^) values. The blindfolding results for the latent variables showed that all endogenous latent variables can be used to provide accurate predictions. The validity power was estimated using the goodness-of-fit (GoF) value. The validity power results for the study model suggest an oversized fit for the study model.

### 5.5 Additional analyses

Robustness tests were conducted to evaluate the reliability of the results. This study used different strategies to verify whether they resulted in substantial differences in results. This study conducted an additional analysis using a logistic regression approach, logistic regression estimations with robust standard errors, and logistic regression estimations using a bootstrapping technique with 5000 replications.

The results in Table 6 demonstrate that, even when additional analyses are used, the results are not substantially different from those of the previous multivariate analyses using the PLS algorithm. The key variables CL and ESG are at the same level of statistical significance and move in the same direction, which is consistent with the prior results. The aforementioned results were in accordance with the data displayed in Table 5. Accordingly, the results confirm the validity and integrity of the predictions.

**Table 6:** Additional analyses.

Independent variables	Logistic regression	Logistic regression (Robust)	Logistic regression (Bootstrap)
CL	−3.409* (0.575)	−3.409* (0.922)	−3.409* (1.149)
ESG	−1.806* (0.478)	−1.806* (0.545)	−1.806* (0.663)
SIZE	1.861* (0.404)	1.861* (0.475)	1.861* (0.667)
LEV	2.732* (0.425)	2.732* (0.678)	2.732* (0.850)
AGE	0.191 (0.549)	0.191 (0.625)	0.191 (0.712)
ROA	−1.282* (0.265)	−1.282* (0.397)	−1.282* (0.423)
COV	0.964* (0.338)	0.964* (0.308)	0.964* (0.335)
SEC	1.386 (1.865)	1.386 (2.069)	1.386 (3.020)
_cons	−8.120 (4.575)	−8.120 (7.760)	−8.120 (10.242)
Industry FE	Yes	Yes	Yes
Year FE	Yes	Yes	Yes

Note: * denote significance at the *1%* level; Standard errors in the model are reported in parentheses. This study used an additional analysis using the logistic regression approach, logistic regression estimations with robust standard errors, and logistic regression estimations using bootstrapping technique with *5000* replications. The variables include financial distress *(FD),* environmental, social, and governance *(ESG),* cost leadership *(CL),* firm *size (SIZE),* firm leverage *(LEV),* firm age *(AGE),* return on assets *(ROA),* coronavirus crisis *(COV),* and sector classification *(SEC).*

## 6 Discussion

Using a sample of U.S. firms from 2016 to 2020, this study explored the connection between a company’s business strategy, ESG performance, and the probability of financial distress. This study employed several techniques to achieve its goals, including the PLS-SEM algorithm, regression analyses, and additional analyses. The findings are as follows: (1) Business strategy significantly and positively influences ESG performance. The results suggest that a firm with a cost leadership strategy has better ESG performance, which is consistent with the theoretical business strategy frameworks [24,25] and theories of contingency, evolutionary, resource-based, stakeholder, institutional legitimacy, signaling, and agency, as well as prior literature findings [26,27,76,77]. (2) Business strategy significantly and negatively influences bankruptcy probability. These results suggest that a firm with a better cost leadership strategy is less likely to experience bankruptcy. These findings are compatible with prior theories, theoretical business strategy frameworks [24,25], and literature findings indicating that a firm with a better business strategy is more robust when facing bankruptcy risk [79]. (3) ESG significantly and negatively influences bankruptcy, suggesting that a firm with better ESG performance is less likely to face bankruptcy. These findings are compatible with prior theories, theoretical business strategy frameworks [24,25], and literature findings indicating that better ESG performance leads to a lower risk of insolvency [37,45,46]. (4) ESG performance negatively affects the probability of bankruptcy through a cost-leadership strategy. This finding indicates that ESG performance is an essential factor that cannot be ignored when mitigating the bankruptcy probability. These findings are compatible with prior theories, theoretical business strategy frameworks [24,25], and the literature indicating that better ESG performance leads to a lower risk of insolvency [37,45,46]. (5) The mediating effect model analysis shows that financial performance negatively affects the probability of bankruptcy through a cost leadership strategy, thus strengthening the negative relationship between business strategy and the probability of bankruptcy. These findings are compatible with previous theories, theoretical business strategy frameworks [24,25], and the literature, which indicate that better performance leads to a lower risk of insolvency [29,37,45,46,83]. (6) Financial performance negatively affects the bankruptcy probability through ESG performance. This finding indicates that financial performance is an essential factor that cannot be ignored when mitigating the bankruptcy probability. These findings are compatible with prior theories, theoretical business strategy frameworks [24,25], and the literature, which indicate that better performance leads to a lower risk of insolvency [29,37,45,46,83].

## 7 Conclusion and policy recommendations

### 7.1 Theoretical implications

From a theoretical perspective, this study makes three significant contributions. First, a firm with better business cost leadership strategy demonstrates higher ESG performance and is less likely to experience financial distress. Accordingly, we expect that firms' decision-makers will have long-term strategic visibility, fully recognize the long-term usefulness of optimal cost leadership strategy, invest in ESG practices, improve their willingness to enhance their tactics and ESG performance, vigorously respond to the call for appropriate policies and procedures, maximize the use of the continuous improvement process to enhance their strategy and ESG performance, reduce risks, and establish a good market image. Second, ESG and financial performance mediate the connection between business strategy and bankruptcy probability, indicating that they are essential factors that cannot be ignored when mitigating bankruptcy probability through a sound business strategy. Finally, financial performance is a mediating explanatory variable in the relationship between ESG performance and bankruptcy probability, indicating that it is an essential factor that cannot be ignored when mitigating bankruptcy probability through sound ESG practices. Consequently, we highlight the need to consider antecedents of the likelihood of financial distress, which should receive more attention in academic research. In this regard, firms must pay attention to the continuous improvement process while taking appropriate measures to educate and motivate employees to achieve best practices.

### 7.2 Managerial contributions

From a managerial perspective, this study proposes the following directions: (1) Firms must pay attention to the importance of continuous improvement while taking appropriate measures to educate and motivate employees to achieve best practices. This leads to an improvement in the internal environment of firms and the work system, which ultimately enhances the performance of firms, whether operational, financial, environmental, or social. (2) Firms' decision-makers should have long-term strategic visibility, fully recognize the long-term benefits of an optimal cost leadership strategy, invest in ESG practices, improve their willingness to enhance their plans and ESG performance, and respond vigorously to the continuous improvement process to enhance their ESG strategies, reduce risks, and establish a good market image. (3) Firms' decision-makers should pay attention to adopting a combination of guidance and supervision, establish a sound “post-evaluation” mechanism for current business strategies and ESG performance, promptly evaluate the effects of strategy implementation, and make corresponding adjustments to correct any deviations in performance, which would assist firms in accomplishing the best practices. (4) Firms' decision-makers should pay attention to the influence of business strategy and the quality of internal controls on their performance and financial safety. Therefore, firms should reinforce their cognition of the construction of better internal control systems to enhance corporate governance performance as an important component of the ESG criteria. (5) Governments should be aware of the importance of actively guiding and effectively supervising firms to enhance their internal control systems and business strategies. (6) Governments should reconsider laws and regulations that enhance the external environment for firms, which would assist firms in fulfilling their ESG responsibilities. (7) Governments should consider financing constraints, enhance tax policies and procedures to support firms' sustainability initiatives, and facilitate financial support for ESG strategies to enable firms to improve their performance.

### 7.3 Limitations and future research perspectives

This study has certain limitations, which give rise to future research areas. This study only adopted a sample of listed firms in the U.S. and did not study firms in other countries. Therefore, future research could be enhanced in the following ways. (1) Exploring more firms within the scope of this study and in other countries. (2) Exploring other factors such as differentiation and focus strategy, working capital management, intellectual capital efficiency, earnings management, and business ethics, which are potential elements to be investigated in financial distress studies.

## Author contribution statement

Ahmed Mohamed Habib: Conceived and designed the experiments; Performed the experiments; Analyzed and interpreted the data; Contributed reagents, materials, analysis tools or data; Wrote the paper.

## Data availability statement

Data will be made available on request.

## Declaration of competing interest

The authors declare that they have no known competing financial interests or personal relationships that could have appeared to influence the work reported in this paper
